# Role of the PD-1/PD-L1 Pathway in Experimental *Trypanosoma cruzi* Infection and Potential Therapeutic Options

**DOI:** 10.3389/fimmu.2022.866120

**Published:** 2022-06-23

**Authors:** Yanina Arana, Rosa Isela Gálvez, Thomas Jacobs

**Affiliations:** Protozoa Immunology, Bernhard Nocht Institute for Tropical Medicine, Hamburg, Germany

**Keywords:** *Trypanosoma cruzi*, co-inhibitory receptors, Chagas Disease, PD-L1, PD-1, Tim-3.

## Abstract

Chagas disease (CD) is a neglected chronic infection caused by the protozoan parasite *Trypanosoma cruzi* (*T. cruzi*). A significant portion of infected people develops cardiac or digestive alterations over a lifetime. Since several chronic infections associated with antigen persistence and inflammation have been shown to lead to T cell exhaustion, new therapies targeting co-inhibitory receptors to regain T cell activity are under consideration. This study explored immune therapeutic approaches targeting the inhibitory PD-1/PD-L pathway in an experimental model for CD. Infected PD-L1 knockout mice (PD-L1 KO) showed increased systemic parasitemia in blood although no significant differences in parasite load were observed in different organs. Furthermore, we found no significant differences in the frequency of activated T cells or proinflammatory cytokine production when compared to WT counterparts. PD-L1 deficiency led to the production of IL-10 by CD8^+^ T cells and an upregulation of Tim-3 and CD244 (2B4). Unexpectedly, the lack of PD-L1 did not contribute to a significantly improved T cell response to infection. Single blockade and combined blockade of PD-1 and Tim-3 using monoclonal antibodies confirmed the results observed in infected. PD-L1 KO mice. Our results describe for the first time that the interruption of the PD-1/PD-L1 axis during acute *T. cruzi* infection does not necessarily enhance the immune response against this parasite. Its interruption favors increased levels of parasitemia and sustained upregulation of other co-inhibitory receptors as well as the production of regulatory cytokines. These results suggest that the clinical application of immune therapeutic approaches targeting the *PD-1/PD-L1* axis in CD might be risky and associated with adverse events. It highlights that more research is urgently needed to better understand the immune regulation of T cells in CD before designing immune therapeutic approaches for a clinical context.

## Introduction

The protozoan parasite *Trypanosoma cruzi* (*T. cruzi*) is the etiological agent of Chagas disease (CD). This chronic disabling disease is endemic in 21 Latin American (LA) countries, where approx. 6 million people are estimated to be infected and 70 million live at risk of contracting infection according to the WHO (1). Migratory movements from LA have spread this disease to all continents and it has become a serious health problem in countries like the US and Spain. There is no vaccine licensed, and the treatments accessible today exhibit severe side effects (2, 3). The natural mechanism of transmission is the direct contact with the contaminated feces released from the vector, a hematophagous triatomine bug, after a blood meal. The infective trypomastigotes invade the host skin or mucosa starting the acute phase, a period of intracellular proliferation and systemic dissemination accompanied by increased parasite numbers in blood and tissues. Although intense inflammatory reactions and immune activation occur, patients might not necessarily show defined clinical symptoms (4, 5). This in consequence leads ultimately to an underdiagnosis of this infection. The adaptive immune response controls the acute infection in most cases but fails to eliminate the parasites leading to the establishment of asymptomatic chronic infection. Several years later, about 30 % of the infected individuals become symptomatic developing pathologies associated with cardiac or digestive tissues, as well as mixed forms, which untreated can lead to death (6). It has been demonstrated that CD4^+^ and CD8^+^ T cells play a crucial role in the control of acute and chronic *T. cruzi* infection (5, 7, 8). Furthermore, *T. cruzi* has developed several strategies to evade immune responses. It has been demonstrated that during the acute phase, lymphocyte activation is suppressed by the production of immune-modulatory molecules (i.e. GPI-anchored mucins, *trans*-sialidases) (9), as well as the inhibition of IL-2R expression (10) promoting immunosuppression that contributes to the spread of the infection. Recently the concept of T cell exhaustion has arisen and been studied in several models of chronic viral infection (11, 12) and several studies have extended this concept to infectious diseases due to intracellular protozoa such as *Toxoplasma gondii*, *Leishmania major*, *Plasmodium* spp., and *T. cruzi* (13–19). Therefore we asked if the increased expression of co-inhibitory receptors could be an additional escape mechanism during acute *T. cruzi* infection. Several studies have demonstrated that co-inhibitory receptors, especially the *PD-1/PD-L1* pathway plays a central role in regulating T cell exhaustion. Its blockade reinvigorates exhausted CD8^+^ T cells, leading to a reduced pathogen burden (11, 20). However, the impact of the PD-1/PD-L1 pathway in acute *T. cruzi* infection is still controversial. Previous studies have shown that *T. cruzi* can modulate the expression levels of co-inhibitory receptors such as PD-1 during experimental infection (5, 8, 21). However, many of these observations were collected from *ex vivo* experiments where the infection models employed different parasite and mice strains with conflicting results. Here, we evaluated the role of the PD-1/PD-L inhibitory pathway during infection with the *T. cruzi* Tulahuen strain to unveil potential intervention points and therapeutic strategies to increase parasite clearance and avoid a progression to the chronic phase. The T cell response was evaluated in PD-L1 KO mice and subsequently, a single blockade and a combined blockade of PD-1 and TIM-3 using monoclonal antibodies were applied as a potential therapeutic intervention in WT mice. We demonstrate that the interruption of the PD-1/PD-L pathway neither reduces parasitemia nor improves the outcome of *T. cruzi* infection. Contrary to our expectations, its interruption favors a higher parasitemia and a pronounced induction of other co-inhibitory receptors like Tim-3 and CD244. Additionally, it induces the secretion of the anti-inflammatory cytokine IL-10.

In conclusion, our data provide evidence that despite the upregulation of PD-1 and its receptor PD-L1, this immune regulatory pathway does not limit the protective immune response against *T. cruzi* infection.

## Materials and Methods

### Mice

7-8 weeks old C57BL/6J (WT) and PD-L1KO on the C57BL/6J background mice were bred under specific pathogen-free conditions at the BSL-3 animal facility at Bernhard Nocht Institute for Tropical Medicine (BNITM), Hamburg. Mice were infected with *T. cruzi* by intraperitoneal (i.p.) inoculation of 2 x 10^3^ bloodstream trypomastigotes diluted in 200 μL of DPBS (PAN-BIOTECH), obtained from infected passage mice. Control mice received 200 μL of DPBS alone. To monitor parasitemia during infection, 2 μL of blood samples were taken from tail vein puncture at the indicated time points. Parasites were counted using a Neubauer chamber (0.02 mm thickness). Mice were euthanized by CO_2_ inhalation and a subsequent neck dislocation.

### Parasites

*In vivo* passage of *T. cruzi* Tulahuen strain was achieved by i.p. inoculation of mice with 5 x 10^5^ bloodstream trypomastigotes resuspended in 200 μL of DPBS. Periodic passages took place every 15 days. For *in vitro* experiments, cell culture-derived *T. cruzi* trypomastigotes were obtained from the supernatant of infected 86Hg39 cells (BNITM) maintained in complete RPMI 1640 medium (PAN-BIOTECH) supplemented with 10 % of fetal calf serum (PAN-BIOTECH), 1 % L-Glutamine (PAN-BIOTECH), and 0.5 %, Gentamycin sulfate (PAA) at 37 °C and 95 % CO_2_ after 3-4 days post-infection.

### Generation of Bone-Marrow-Derived Dendritic Cells

Hematopoietic stem cells from the bone marrow were isolated under sterile conditions and 3 x 10^6^ cells were seeded with a complete BMDCs medium composed of DMEM, 10 % fetal calf serum, 1 % L-Glutamine, 0.5 %, Gentamycin, and 10 % GM-CSF. On days three and six after bone marrow isolation, 8 mL of complete BMDCs medium were added additionally to the culture. On day seven, BMDCs were fully differentiated and used for further experiments. For PD-L1 expression analysis, BMDCs were co-incubated in a ratio of 1:1 *T. cruzi* trypomastigote per cell for 6 hours. Afterward, cells were washed twice, resuspended in a complete BMDCs medium, and incubated for 72 hours. Next, the BMDCs were stained with fluorescent antibodies against surface markers before fixation. BMDCs were then permeabilized (eBioscience Foxp3/Transcription Factor Staining Buffer Set) and stained for intracellular *T. cruzi* with anti-*T. cruzi* polyclonal rabbit antiserum in a 1:200 dilution (BNITM). PD-L1 expression was evaluated by flow cytometry and BMDCs isolated from PD-L1 KO mice were included as an internal control of PD-L1 expression.

### Organ Sampling

Mice were sacrificed at different time points post-infection as indicated in the figures. To analyze parasitic load, 25 mg tissue samples of heart, liver, skeletal muscle and 10 mg spleen were harvested, and rinsed in DPBS, to avoid contamination with blood parasites. Samples were stored in liquid nitrogen until DNA isolation. For flow cytometry, the spleen was harvested and lymphocytes were isolated. Briefly, the spleens were collected at 4°C in RPMI, mashed through a 70-μm-pore-size cell strainer, and centrifuged at 315 g for 5 min at 4°C. After that, RBC lysis was done for 5 min at RT. Subsequently, the cells were centrifuged and washed again, then resuspended in 10 mL sterile complete RPMI 1640 medium and passed through a 40-μm-pore-size cell filter. Finally, the cell number was determined.

### Flow Cytometry Analysis

3 x 10^6^ cells were used for surface and intracellular staining. Briefly, splenocytes were incubated with the antibody cocktail diluted in Fc-block at 4 °C and for 30 min in the dark. After surface antibody incubation cells were washed. For intracellular staining, cells were stimulated with 50 ng/mL PMA (SIGMA) and 500 ng/mL Ionomycin (SIGMA) for six hours at 37°C and 5 % CO_2_, and 2 μM Monensin (BioLegend) was added during the last 5 hours of culture. Cells were washed twice with cold DPBS (315 g, 5 min, and 4°C), and stained with antibodies directed against surface antigens (as described above). In the next step, the cells were fixed and permeabilized according to the manufacturer’s protocol of the Foxp3/Transcription factor staining buffer set. Briefly, after surface staining, cells were washed twice with cold DPBS and fixed with 100 μL fixation buffer for 30 min at RT. After that cells were washed twice with permeabilization buffer and stained with fluorescently labeled anti-cytokine antibodies diluted in permeabilization buffer for 30 min at RT. After incubation, cells were washed twice with permeabilization buffer and suspended in 200 μL of FACS buffer (1 % FCS, 0.1 % sodium azide in PBS) for fluorescence measurements on a BD LSRII flow cytometer (BD, Biosciences, Heidelberg). FMO controls were used for gating. A complete list of antibodies used is given as **Supplementary Material** in **Table 1**. Cytometry data was analyzed using FlowJo 10.8.1.

### Cytometric Beads Assay-LEGENDplex™

Cytokine profile was determined using the LEGENDplex™ Mouse Th Cytokine Panel (13-plex). Serum samples were processed following the manufacturer’s instructions, afterwards, the samples were measured with the Accuri C6 cytometer (Accuri Cytometer Inc., Ann Arbor).

### *In Vivo* Blocking Assays

For PD-1 blockade, 0.2 mg of anti-PD-1 mAb (RMP1-14, BioLegend) was administered i.p. at the time of infection and a second dose, 7 days post-infection. Control mice were administered with the same amount of rat IgG2a isotype control (RTK2758, BioLegend). For blockade of PD-1 and TIM-3, 0.2 mg of anti-PD-1 mAb and 0.2 mg of anti-TIM-3 (RMT3-23, BioLegend) were administered following the same scheme as applied for the PD-1 blockade. The control group received 0.2 mg of rat IgG2a isotype control.

### Parasite Detection With Quantitative Real-Time PCR

Frozen tissue samples were mechanically disrupted and homogenized. The tissue suspension was incubated overnight at 56 °C with lysis buffer and Proteinase K from QIAamp DNA Mini Kit (QIAGEN) and DNA isolation was performed according to manufacturer specifications. The concentration of DNA was determined using a NanoDrop 2000 Spectrophotometer (PeqLab/Thermo Scientific). The standards for the qPCR were generated by spiking tissue homogenates from naive mice to which 10^5^ cell-cultured *T. cruzi* trypomastigotes were added. DNA was isolated as mentioned above and serially diluted with 25 μg/mL DNA isolated from unspiked naive mice tissue. The 10-fold dilution series contained DNA from 10^5^ to 10^-2^ parasites, equivalents per 50 ng of total DNA. A standard curve was generated from these standards, in triplicate reactions, to determine the parasitic load in the organs of infected mice. Real-time PCR Mastermix was prepared using the QuantiTec SYBR Green PCR Kit (QIAGEN) and run on a Rotor-Gene (R Corbett Research). Primers target the minicircle variable region from kDNA and amplify a 330 bp fragment. The amount of T. cruzi from kinetoplast DNA (kDNA) was quantified in the mouse-GAPDH housekeeping gene. Samples were analyzed by duplicates. Primer sequences were as follows: Tc 121F 5´–AAATAATGTACGGGKGAGATGCATGA-3´, Tc 121 R 5´–GGTTCGATTGGGGTTGGTGTAATATA -3´, GAPDH-F 5´-GTCGGTGTGAACGGATTTGG-3’, and GAPDH-R 5’-TTCCCATTCTCGGCCTTGAC-3’. Thermal Profile: Initial _DNA denaturation_ = 95°C, 900 sec; (T_DNA denaturation_ = 94°C, 60 sec; T_primer annealing_ = 68°C, 60 sec; T_elongation_ = 72°C, 60 sec) x 5; following by (T_DNA denaturation_ = 94°C, 45 sec; T_primer annealing_ = 64°C, 45 sec; T_elongation_ = 72°C, 45 sec) x 40; Final _elongation_ = 72°C, 600 sec. A melting curve phase program was applied with the continuous measurement between 62°C and 95°C. Duplicate values for each DNA sample were averaged (geometric mean) and parasite equivalent load was calculated automatically using the Rotor-Gene 6000 Series Software 1.7 (Corbett research/Qiagen) Briefly, we plotted the Ct value against each standard of known concentration and calculated the linear regression line of this curve. To normalize the amount of DNA, GAPDH was used to correct the initial sample mount. Murine GADPH and Tc121/122 amplification have the same efficiency. The parasite loads below the limit of quantification (LOQ), which means less than 1 parasite equivalent, were set to LOQ/2 (0.05 parasite equivalents per 50 ng of DNA), as previously described in (22, 23).

### Statistical Analysis

Data were analyzed for normal distribution before running statistics. Statistical analyses were performed in GraphPad Prism 9.3.0. If not otherwise stated in the legend, the Kruskal-Wallis test was used to determine statistical differences followed by a pairwise comparisons analysis using Dunn’s test. Non-significant results (p>0.05) were not mentioned or plotted to avoid busy figures. All data are shown as mean ± SEM.

## Results

### *T. cruzi* Infection Induces Expression of PD-L1 on Antigen-Presenting Cells

**Figure 1:**
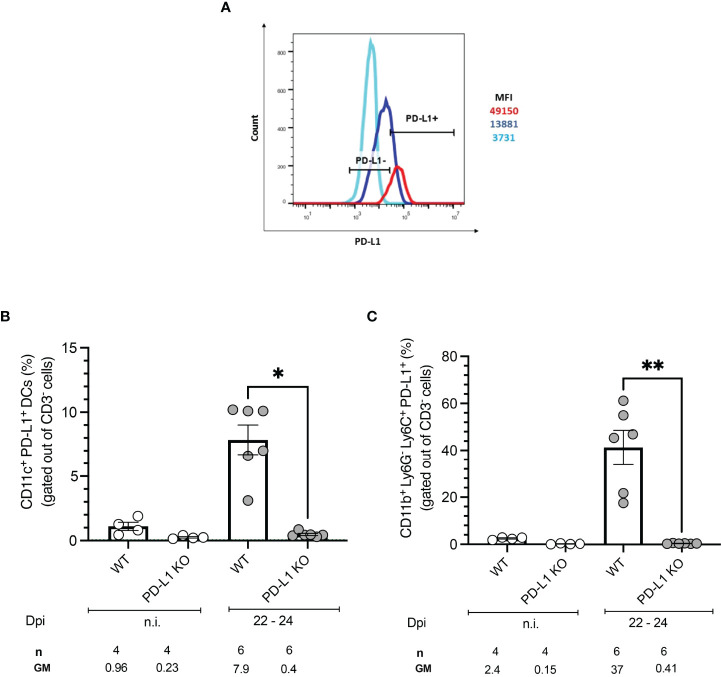
PD-L1 expression on antigen-presenting cells after *in vitro* and *in vivo* infection with *T. cruzi.* BMDCs were infected with *T. cruzi in vitro.* After 72 h the PD-L1 Expression was analyzed by flow cytometry. **(A)** The Histogram shows the MFI of PD-L1 expression on infected (red) and non-infected (blue) BMDCs. FMO staining control (light blue) was used to identify and gate the PD-L1cells population. Expression of PD-L1 on APCs from WT and PD-L1 KO mice infected with *T. cruzi.* On day 22- 24 post-infection spleens from infected and n.i. mice were isolated and the expression of PD-L1 was evaluated by flow cytometry. **(B)** Expression of PD-L1 on CD11b^-^ CD11c^+^ (DCs). **(C)** Expression of PD-L1 on CD11b^+^ Ly6G^-^Ly6C^+^ MΦ. Data shown are corresponding to two independent experiments n.i. = 4; infected =6. Asterisks denote P values of <0.05. *P<0.05 and **P<0.01.

To test whether *T. cruzi* infection induces expression of the PD-1 ligand PD-L1 on antigen-presenting cells (APCs), BMDCs from WT mice were infected *in vitro* with *T. cruzi* trypomastigotes and PD-L1 expression was evaluated by flow cytometry. The experimental setup, gating strategy, and representative dot plots are depicted in **Figures S1A, B**. CD11c and CD86 expression were employed to define BMDCs and infected cells were identified by staining with an anti-*T. cruzi* polyclonal rabbit antiserum. Results showed a clear upregulation of PD-L1 on BMDCs infected with *T. cruzi* compared to non-infected cells during *in vitro* infection (**Figure 1A**). Specific induction of PD-L1 after *T. cruzi* infection *in vivo* was measured at 22-24 days post-infection (dpi). The gating strategy and representative dot plots are depicted in **Figure S2A**. For this analysis, spleens were harvested and antigen-presenting cells were characterized by flow cytometry. Dendritic cells (DCs) were defined as CD3^-^CD11b^-^CD11c^+^ while classical macrophages (MΦ) were defined as CD3^-^CD11c^-^C11b^+^Ly6C^+^. Infected WT mice showed an 8-fold higher frequency of PD-L1^+^ DCs than those from non-infected (n.i.) WT mice (**Figure 1B**). WT mice had a significantly higher frequency of PD-L1^+^MΦ after infection, in mean 37% PD-L1^+^MΦ were found, which is 15-fold more compared to n.i. WT counterparts (**Figure 1C**). As expected DCs and MΦ from PD-L1 KO mice did not express PD-L1. We also analyzed the expression of PD-L2 to prove the possibility of a compensatory role of this ligand. Our results revealed a low frequency of PD-L2^+^ DCs in n.i. WT and PD-L1 KO mice. The infection led by trend to a small increase of PD-L2^+^ DCs in both mice strains which was not statistically significant (**Figure S2B**). However, the PD-L2 expression increased statistically significantly on CD11b^+^ Ly6C^+^ Ly6G^-^ MΦ from WT mice at 22-24 dpi (**Figure S2C**). These data show that in our model infection with the *T. cruzi* Tulahuen strain can induce PD-L1 expression on host cells.

### Co-inhibitory Receptors PD-1, Tim-3, and CD244 Are Expressed on T Cells During Acute *T. cruzi* Infection *in vivo* and PD-L1 Deficiency Reinforces Their Expression

We next investigated if *T. cruzi* infection modulates the expression of co-inhibitory receptors *in vivo*. WT mice were infected and sacrificed at two different time points: 10 to 15 dpi and 22 to 24 dpi. Spleens were harvested, and CD4^+^ and CD8^+^ T cells were further characterized by flow cytometry. Representative plots and the gating strategy are depicted in **Figures S3A, B**. Infected WT mice displayed significantly higher frequencies of PD-1^+^ CD4^+^ T cells at the beginning of the acute phase (10 -15 dpi) compared to n.i. mice and also compared to PD-L1KO mice. We observed that

PD-1 expression in the WT mice was only transient since at the peak of parasitemia at 22-24 dpi the PD-1 expression had already decreased to levels comparable to n.i. mice, while their PD-L1 KO counterparts strongly upregulated the expression of

**Figure 2:**
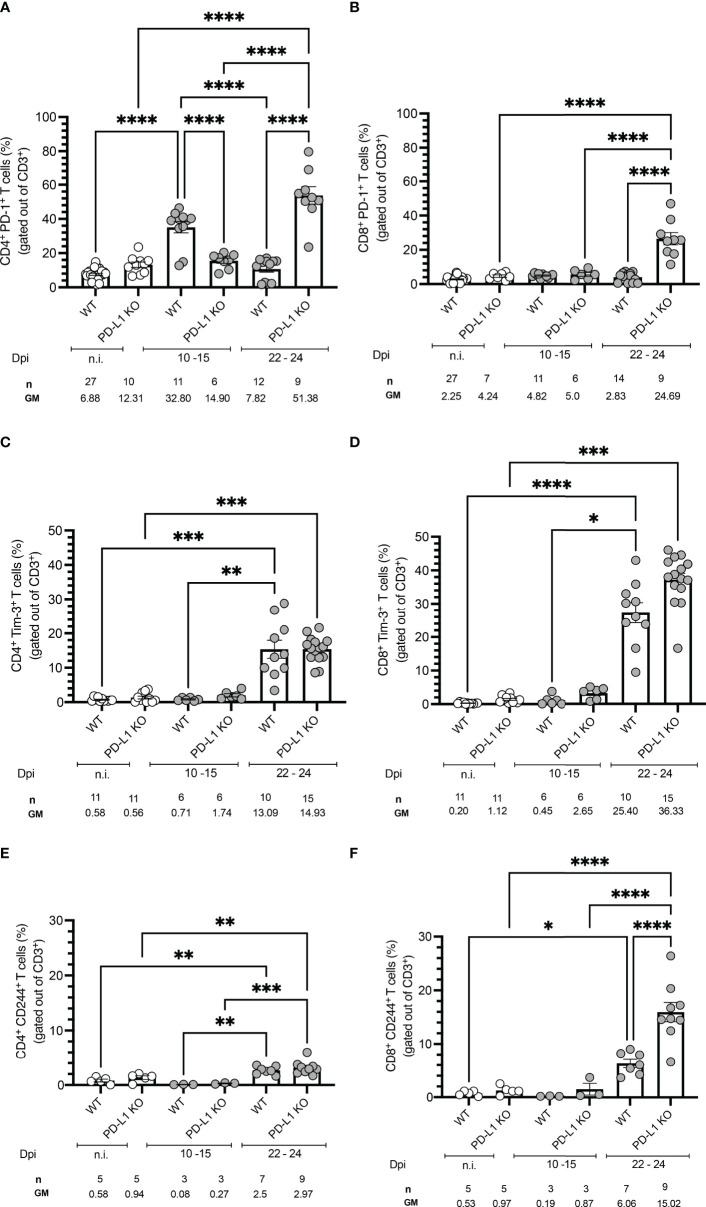
Time course of co-inhibitory receptors expression on T cells from WT and PD-1 KO mice infected with *T. cruzi.* Time-course experiments showing expression of **(A)** PD-1 on CD4^+^ and **(B)** CD8^+^ T cells; **(C)** Tim-3 on CD4^+^ and **(D)** CD8^+^ T cells; **(E)** CD244 on CD4^+^ and **(F)** CD8^+^ T cells. Error bars indicate standard errors of the means (SEM). Data from three independent experiments. Under the graphs, n is the absolute number of mice used per time point, and the values below are the geometric means (GM). Data were analyzed for statistical significance using the Kruskal-Wallis test following Dunn´s multiple comparisons test. Asterisks denote P values as described in Methods; ns (not significant) was not plotted to avoid a busy figure.

PD-1 (51.8 % of all CD4^+^ T cells) towards the end of the acute stage (**Figure 2A**). The upregulation of PD-1 on CD8^+^ T cells was not as dynamic as on CD4^+^ T cells, since PD-1 expression at 10-15 dpi did neither increase in WT mice nor PD-L1 KO mice. In WT mice the PD-1 expression also did not increase at the peak of parasitemia. Only at 22-24 dpi, the frequency of PD-1^+^CD8^+^ T cells increased in PD-L1KO mice (**Figure 2B**). These results demonstrate that *T. cruzi* infection induces PD-1 on CD4^+^ T cells from WT and PD-L1 KO mice. CD8^+^ T cells neither in WT nor in PD-L1 KO mice respond to infection with the upregulation of PD-1. Only in infected PD-L1 KO mice did the expression of PD-1 on CD8^+^ T cells strongly increased. Next, we evaluated, if the expression of Tim-3 was induced after *T. cruzi* infection and if it was affected by the interruption of PD-1/PD-L1 signaling in PD-L1 KO mice. The results in **Figure 2C** for CD4^+^ T cells and **Figure 2D** for CD8^+^ T cells showed no significant increase in

Tim-3 expression in WT or PD-L1KO mice in comparison to n.i. mice at 10-15 dpi. However, on 22-24 dpi Tim-3^+^CD4^+^ T cells increased in WT and PD-L1KO mice to a comparable extent (13.09 % and 14.93 % respectively) but the increase of

Tim-3^+^ CD8^+^ T cells was more pronounced, reaching 25.40 % in WT mice and 36.33 % in PD-L1 KO mice. These results confirmed that in this acute infection model, Tim-3 might represent a compensatory regulatory mechanism for the disrupted

PD-1/PD-L1 pathway, mainly affecting CD8^+^ T cells. Finally, we aimed to evaluate the expression of CD244 (2B4). CD244 is found on many immune cells but mainly on NK cells and activated CD8^+^ T cells. The contribution to the effector function of T cells needs further investigation since it is not completely clarified if it acts co-stimulatory or co-inhibitory (20, 24). In the context of parasitic diseases, it has been barely explored. Since only one report analyses CD244 function and shows an upregulation in the chronic human CD (25), we wanted to elucidate if CD244 represents a potential regulatory pathway in the acute stage of infection. In **Figure 2E** the expression of CD244 on CD4^+^ and in **Figure 2F** on CD8^+^ T cells is depicted. The kinetic of CD244 expression followed a similar pattern as the Tim-3 expression, meaning that at

10-15 dpi neither WT nor PD-L1 KO mice upregulated this receptor in comparison to n.i. mice. The expression of CD244 increased significantly on CD4^+^ T cells in WT and PD-L1 KO mice at 22-24 dpi. The induction of CD244 was also found on CD8^+^ T cells at 22-24 dpi in WT mice and even stronger and highly statistically significant in PD-L1 KO mice. These results support the idea of a compensatory pathway *via* CD244 after disruption of PD-1/PD-L1 signaling.

### PD-L1 Deficiency Does Not Affect Activation, IFN-γ Production, or Granzyme B Production but Induces IL-10 Secretion by CD8^+^ T Cells

**Figure 3:**
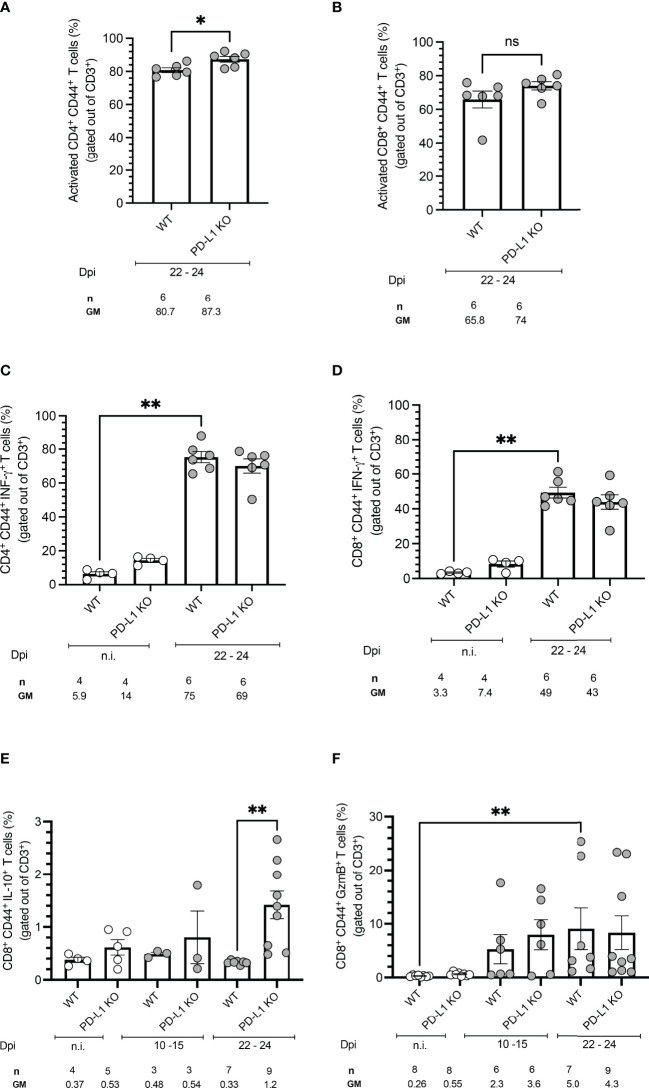
PD-L1 deficiency does not affect activation, IFN-γ production, or Granzyme B production but induces IL-10 secretion on CD8^+^ T cells after stimulation. Spleen cells were isolated from mice and analyzed by flow cytometry after a 5 h stimulation with PMA/Ionomycin. **(A, B)** PD-L1 deficiency does not affect T cell activation, defined by CD44 expression, after *T. cruzi* infection on 22-24 dpi. **(C, D)** *T. cruzi* infection induces IFN-γ on CD4^+^ and CD8^+^ T cells from WT and PD-L1 Ko mice. PD-L1 deficiency does not affect IFN-γ production. **(E)** IL-10 is only induced at 22-24 dpi in CD8^+^ T cells from PD-L1KO Mice **(F)** Granzyme B is significantly upregulated only on CD8^+^ T cells from WT mice. PD-L1 deficiency does not affect Granzyme B production. Error bars indicate standard errors of the means (SEM). Data from two independent experiments. Under the graphs, n is the absolute number of mice used per time point, and the values below are the geometric means (GM). Data were analyzed for statistical significance using the Kruskal-Wallis test following Dunn´s multiple comparisons test. Asterisks denote P values as described in Methods; ns (not significant) was not plotted to avoid busy figures.

To evaluate an early effect of PD-L1 deficiency in the control of acute *T. cruzi* infection by CD4^+^ and CD8^+^ T cells, splenocytes from WT mice and PD-L1 KO mice were isolated and stimulated with PMA/Ionomycin. Functionality was measured by analysis of IFN-γ and IL-10 production. Cytotoxicity was evaluated based on Granzyme B production. PD-L1 deficiency did not induce significant differences in the frequency of activated CD4^+^ and CD8^+^ T cells in comparison to infected WT mice (**Figures 3A, B**). The *T. cruzi* infection led to a strong increase in IFN-γ production by CD4^+^ and CD8^+^ T cells. The capability to produce IFN-γ was not affected by PD-L1 deficiency (**Figures 3C, D**). The results in **Figure 3E** show that PD-L1 deficiency is accompanied by increased production of IL-10 by CD8^+^ T cells at 22-24 dpi in comparison to infected WT mice. Granzyme B expression only slightly increased at 10-15 dpi in both WT and PD-L1 KO mice without reaching statistical significance. At 22-24 dpi only WT mice showed a significant increase of Granzyme B in comparison to n.i. counterparts (**Figure 3F**).

### No Effect of Combined Therapy by Monoclonal Antibodies Against PD-1 and Tim-3 During *T. cruzi* Infection *In Vivo*

**Figure 4:**
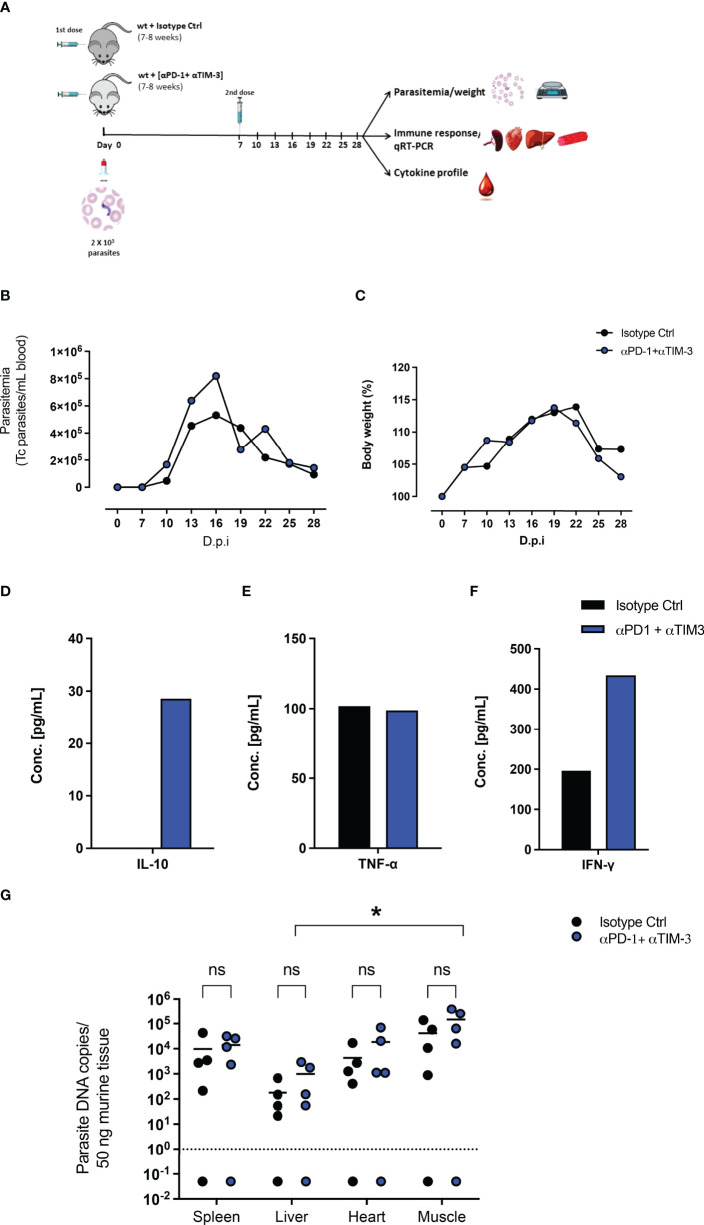
Combined therapeutic intervention with αPD-1 and αTim-3 monoclonal antibodies does not reduce parasitemia but induces IL-10. **(A)** Scheme of infection and combined αPD-1 and αTim-3 therapy. Mice were infected i.p. with 2x10^3^ T. cruzi parasites on day 0. They received αPD-1 and αTim-3 in a concentration of 0.2 mg each/dose on day 0 and day 7 after infection. The control group received i.p. 2 doses of isotype antibody in the same concentration. WT mice infected and isotype treated n=8; WT infected and αPD-1+αTIM- 3 n=7. Results from two independent experiments **(B)** Parasitemia and **(C)** body weight were analyzed over the course of infection (28 days) and are shown as means with SD. Whole blood samples were collected, and sera were isolated and pooled. Cytokine levels were determined by a cytometric bead assay. Results are expressed as the cytokine concentration of the pooled sera samples **(D)** IL-10, **(E)** TNF-α, and **(F)** IFN-γ. **(G)** Effect of interruption of PD-1/PD-L1 signaling on parasite load analyzed by *T. cruzi*-specific qRT-PCR. Comparative parasite load in spleen, liver, heart, and skeletal muscle from infected and treated with blocking antibodies against αPD-1+ αTIM-3 or their respective isotype controls. Parasite load was calculated from a standard curve. The standard error of the mean is indicated (SEM). Asterisk denotes P values of < 0.05 by One-way ANOVA compared to isotype control values. P < 0.05*; ns (not significant).

Due to the upregulation of PD-1 and Tim-3 on CD4^+^ and CD8^+^ T cells after *T. cruzi* infection and due to their increased induction in PD-L1 KO mice, a combined blockade of PD-1 and Tim-3 was assumed to be a potential therapeutic intervention. On the one hand to increase the functionality of CD4^+^ T cells, unleash cytotoxic mechanisms of CD8^+^ T cells, and thus promote better parasite elimination. On the other hand, it potentially blocks compensatory mechanisms between both pathways. **Figure 4A** shows the experimental setup. WT mice were infected with *T. cruzi* and treated with monoclonal antibodies against PD-1 and Tim-3 or with an isotype control antibody. The results depicted in **Figure 4B** showed that the combined blockade had no impact on the control of the parasitemia in blood. Also, the resistance to the infection was not improved, since no significant differences were observed regarding the bodyweight (**Figure 4C**). To evaluate the effect on the systemic inflammatory response, serum samples were collected and pooled and their cytokine profile was evaluated by a cytometric beads assay. After the combined blockade IL-10 could be detected in the serum of infected mice while it was not detectable in the pooled sera from infected mice treated with the isotype control antibody, confirming the results seen in the infection of PD-L1 KO mice (**Figure 4D**). Furthermore, TNF-α was present in sera from both groups in a comparable concentration, suggesting not to be affected by the combined blockade (**Figure 4E**). Using this experimental approach, it was possible to detect IFN-γ in pooled sera from infected mice (200 pg/mL) and the combined blockade induced a notable increase of systemic IFN-γ (> 400 pg/mL) (**Figure 4F**). Finally, we evaluated the effect of the combined blockade on the tissue parasite load during *T. cruzi* infection. For this, tissue samples from the spleen, liver, heart, and skeletal muscle corresponding to the infected mice treated with blocking antibodies and their respective isotype controls were isolated and the parasite load was evaluated by qRT-PCR. The results shown in **Figure 4G** do not exhibit significant differences in the parasite load in the analyzed tissues between infected mice that received a combined blockade and their respective isotype control-treated mice. However, by trend, an increased parasite load was observed in the group that received the combined blockade. Parasitemia in skeletal muscle was significantly higher than the parasitemia found in the liver tissue.

## Discussion

After acute infection with *T. cruzi*, which is associated with a high antigen load and production of pro-inflammatory cytokines, antigen-specific T cells, especially CD8^+^ T cells, reduce the pathogen load. However, the immune response often fails to clear residual pathogen reservoirs in a specific tissue leading to chronic infection. Chronic antigen exposure is accompanied by a gradual loss of T cell effector function leading ultimately to T cell exhaustion (11, 12). In various settings, it was already shown that exhausted T cells expressing PD-1 can be revigorated, at least in part, by blocking the PD-1/PD-L1 pathway (20). Therefore, manipulation of this pathway might pave new avenues for the treatment of parasitic infections some of which are known to persist lifelong.

The importance of the PD-1/PD-L inhibitory pathway has been studied previously in the context of *T. cruzi* induced acute myocarditis (5) and a chronic model of Chagas heart disease in mice (8). Whereas the first publication reported an increased cardiac inflammatory response, reduced blood parasitemia, and cardiac parasitism, along with high mortality rates upon PD1/PD-L blockade; the second showed that

PD-1/PD-L1 blockade together with an immunization using irradiated *T. cruzi* only decreased the blood parasitemia but did not affect cardiac parasite load nor cytokine production in the heart tissue. These conflicting results reflect the major problem in CD research. There are up to date no standardized animal models to study *T. cruzi* infection and subsequent CD. Using different isolates and strains of *T. cruzi* parasites introduce a serious bias and differences in the immune response from mice with different genetical background leads to potential different outcomes. Moreover, there is neither a consensus about standardized routes of infection (intra-peritoneal, intravenous, subcutaneous, oral, congenital) nor on the assessment of parameters to characterize the acute and chronic infection or their pathological consequences in the different infected tissues (26–29). Therefore, it is essential to highlight that our study was focused on a model established on the intraperitoneal infection of female C57BL/6 mice with *T. cruzi* trypomastigotes of the reticulotropic *T. cruzi* strain Tulahuen (DTU IIe) (30). Our results are limited to the acute stage of infection until day 28 post-infection since the majority of PD-L1 KO mice succumb to infection after this time point. Trypanosomes have developed several strategies to down-regulate T cell responses, the modulation of the

PD-1/PDL pathway being one of them, that could avoid their complete clearance and allow their persistence (15, 16). Based on this knowledge, we first aimed to address, if *T. cruzi* infection modulates PD-1 expression at the beginning of the infection and if PD-1 expression is necessarily associated with exhaustion of T cells in the acute stage. Second, we aimed to interrupt the PD-1/PD-L1 pathway during the infection using PD-L1 KO mice and subsequently test the therapeutic potential of a therapeutic blockade of PD-1. We demonstrated that PD-L1 expression is upregulated in bone marrow-dendritic cells (BMDCs) after *in vitro* and *in vivo* infection with *T. cruzi*. This confirms previous reports, that used other *T. cruzi* strains (5, 8, 21). This suggests that the induction of PD-L1 is not parasite strain or host-dependent, and emphasizes the involvement of this inhibitory pathway in the context of *T. cruzi* infection. Some common γ -chain (γ c) cytokines (31, 32) and inflammatory cytokines have been described as the driving mechanisms that

up-regulate PD-L1 on APCs or somatic cells in the tissue (32–34). Our results suggest that enhanced levels of PD-L1 observed in infected BMDCs and APCs are associated with the presence of the parasite inside the cells and are not induced by excretory/secretory parasite or cell products since the PD-L1 expression levels on uninfected cells in the same culture remained low and cells incubated with conditioned media from infected cells did not induce PD-L1 (data not shown). These results suggest that *T. cruzi* could induce the PD-L1 expression through activation of other alternative pathways (e.g., signals associated with its intracellular replication). Our results show that infection with *T. cruzi* induces the expression of PD-1 on T cells *in vivo*. Interestingly, we observed statistically significant higher frequencies of CD4^+^ T cells expressing PD-1 during the course of the infection whereas only a slight increase of CD8^+^ PD-1^+^ T cells was found. Our results support the findings observed during the infection by *T. cruzi* Y, a cardiotropic strain (5), and are in contrast to the common finding that PD-1 is mainly induced on CD8^+^ T cells during infections with viral pathogens (35). In protozoan parasite infections, CD4^+^ T cells are critical in the establishment of a Th1 response to activate the microbicidal activity of macrophages and the generation of cytotoxic CD8^+^ T cells that recognize and kill infected cells (36). The up-regulation of PD-1 on CD4^+^ T cells might represent an immunoregulatory mechanism to avoid tissue damage caused by an exacerbated inflammatory response (32, 33). The up-regulation of Tim-3 upon blockade of the PD-1/PD-L1 pathway with anti-PD-1 monoclonal antibodies has been associated with the failure of the therapy in the cancer (37, 38). We observed also a significant up-regulation of Tim-3 on CD8^+^ T cells from infected PD-L1KO mice. Of note, this increase of Tim-3 on CD8^+^ T cells is also common to other *T. cruzi* strains, since using the *T. cruzi* strain Brazil, described as a myotropic strain that can establish chronic disease, the Tim-3 expression at 22 dpi was also significantly higher compared to WT mice (**Figure S4D**). CD244 (2B4) is a co-inhibitory receptor whose expression is up-regulated on antigen-specific CD8^+^ T cells in chronic viral infections (20), and it is also up-regulated on CD8^+^ T cells from chronic Chagas patients with severe forms of the disease (39). Its expression upon PD-L1 deficiency suggests a potential compensatory mechanism of this pathway, that needs to be further analyzed. Interestingly, and in contrast to our expectations, PD-L1 KO mice showed a trend of higher blood parasitemia (**Figure S3B**). This could be due to an impaired parasite clearance and suggests the possibility of a co-stimulatory function for PD-L1. The possibility of a co-stimulatory role for PD-L1 associated with a protective role has been hypothesized for infections with *Listeria monocytogenes* (40) and *Mycobacterium tuberculosis* (41). However, following this hypothesis, it remains striking, that we did not observe a decreased frequency of activated CD4^+^ CD44^+^ T cells or CD8^+^ CD44^+^ T cells in infected PD-L1 KO mice in comparison to infected WT mice. Interestingly it was shown recently that PD-1 derived signals promote an optimal CD8^+^ T cell memory formation. Therefore, disruption of the PD-1/PD-l1 pathway may lead to a decreased control of *T. cruzi* at later stages (42).

Since PD-L2 is an alternative ligand of PD-1 and has been reported to compensate for the PD-L1 deficiency in some infections (43), it could be speculated that also in our model PD-L2 might compensate for the missing signaling *via* PD-L1. Its induction during the *T. cruzi* infection has been reported on T cells and macrophages (5, 21) but a compensatory role has yet not been demonstrated. In our exploratory experiments, there was non-significant upregulation of PD-L2 on APC from PD-L1 KO mice in comparison to WT-infected mice (**Figures S2B, C**). An alternative explanation of the impaired parasite clearance could be an activation of other immunoregulatory mechanisms in the absence of PD-1/PD-L1 signaling. The increased expression of IL-10 might represent such a compensatory mechanism. It has been demonstrated that IL-10, a potent regulatory cytokine, is involved in pathogen persistence during chronic infections (36, 44). Previous studies showed that during the infection by intracellular parasites, including *T. cruzi*, IL-10 acts by down-regulating T-cell responses favoring the parasite persistence (7, 36, 45). Moreover, studies in chronic LCMV infection and Toxoplasma showed that PD-L1 and IL-10 are independent pathways and act in parallel to regulate the immune response preventing immune mediated-tissue damage (46–48). There is strong evidence that IL-10 plays a key role in regulating the expression of the PD-1 ligands (36). Therefore, the interaction between both pathways cannot be discarded. Our results suggest a compensatory relationship between both pathways that might be reflected by the impaired parasite clearance observed during infection of PD-L1KO mice and are supported by reports where PD-L1 blockade promotes the induction of IL-10 (49). Additionally, a recent study on Toxoplasma infection revealed that the absence of PD-1 signaling promotes an increase in IL-10 production by CD4^+^ and CD8^+^ T cells, which increases the susceptibility to other opportunistic infections (14). Based on our results, it would be interesting to evaluate if the blockade of the IL-10/IL-10R pathway influences the level of PD-1 or PD-L1 expression. Due to the upregulation of PD-1 during the course of *T. cruzi* infection and the upregulation of Tim-3 upon PD-L1 deficiency, a combined blockade of PD-1 and Tim-3 using monoclonal antibodies was evaluated in infected *T. cruzi* WT mice. Our results showed that the combined blockade of

PD-1 and Tim-3 did not improve the course of infection. Neither the activation nor the functional capacity of T cells in comparison to infected isotype-treated control mice was found to be increased. Nevertheless, the combined blockade led to an increase of IL-10 and IFN-γ systemically. Thus, our hypothesis, that in *T. cruzi* infection, compensatory signaling through the Tim-3 pathway might be sustaining the potential dysfunction of T cells upon interruption of the PD-1 signaling could not be confirmed after the combined blockade experiments. The qRT-PCR results showed a trend, but not a statistically significant increase in parasite load in the analyzed tissues. This result is in agreement with a work in *T. cruzi* Sylvio X10/4 chronic infection where it was shown that blocking PD-1/PD-L1 interaction with monoclonal antibodies did not reduce the parasite load in the heart and the parasite persists due to a failure of the blockade to achieve an increased IFN-γ and TNF-α production by infiltrating T cells (8). In summary, our study shows that disruption of the PD-1/PD-L1 inhibitory pathway in the acute phase of the *T. cruzi* infection in mice does not provide increased protection against this parasite. In contrast, the interruption of the PD-1/PD-L1 pathway showed an increase in parasitemia as well as in the production of regulatory cytokines such as IL-10. This cannot be explained by enhanced induction of other co-inhibitory pathways such as Tim-3 in the absence of PD-1, as the dual blockade of these two pathways did not successfully enhance parasite elimination or T cell activation. It is tempting to speculate that other inhibitory pathways might favor persistence. Therefore, our results demonstrate that further research is urgently needed to better understand T-cell regulation in CD before immunotherapeutic approaches can be developed for a clinical setting, since the clinical use of immunotherapeutic approaches targeting the PD-1/PD-L1 axis in CD may be risky and associated with adverse events.

## Data Availability Statement

The raw data supporting the conclusions of this article will be made available by the authors, without undue reservation.

## Ethics Statement

The animal study was reviewed and approved by The office of consumer protection in Hamburg, Germany (Application Nr. 52/17).

## Author Contributions

TJ developed the concept of the study, guided the writing process, and edited the final manuscript. YA and RIG performed the experiments, analyzed the data, and wrote the manuscript; All authors drafted the conclusion and provided critical feedback to shape the manuscript.

## Funding

YA was supported by DAAD (Deutscher Akademischer Austausch Dienst) German Academic Exchange Service with Research Grant for Doctoral Students and Young Academics (500154434) Ref.# 91537601. RIG was a recipient of a Merit-based Ph.D. scholarship from the Konrad Adenauer Foundation (KAS), Germany.

## Conflict of Interest

The authors declare that the research was conducted in the absence of any commercial or financial relationships that could be construed as a potential conflict of interest.

## Publisher’s Note

All claims expressed in this article are solely those of the authors and do not necessarily represent those of their affiliated organizations, or those of the publisher, the editors and the reviewers. Any product that may be evaluated in this article, or claim that may be made by its manufacturer, is not guaranteed or endorsed by the publisher.

## Supplementary Material

Supplementary Figure 1
**(A)** Experimental set-up for the infection of BMDCs *in vitro.* **(B)** Flow cytometry gating strategy for BMDCs.

Supplementary Figure 2
**(A)** Flow cytometry Gating strategy and exemplary dot plots for PD-L1 expression *in vivo* on CD11c^+^ CD11b^-^ cells (DCs) and CD11b^+^Ly6G^-^Ly6C^+^ cells (MΦ) at day 15 post-infection. **(B)** Expression of PD-L2 on CD11c^+^CD11b^-^ (DCs) and, Ly6G-Ly6C+CD11b+ (MΦ). Data are a compilation of two independent experiments (n.i. = 4; infected=6). Data were analyzed for statistical significance using the Kruskal-Wallis test following Dunn´s multiple comparisons test. Asterisks denote P values as described in Methods; ns (not significant) were not plotted to avoid busy figures.

Supplementary Figure 3
**(A)** Gating Strategy and exemplary dot blots for co-inhibitory receptors expression on CD4^+^ and CD8^+^ T cells from n.i. and infected WT and PD-L1 KO mice. **(B)** PD-1 on CD4^+^ T cells **(C)** PD-1 on CD8^+^ T cells. **(D)** Tim-3 on CD4^+^ T cells **(E)** Tim-3 on CD8^+^ T cells. **(F)** CD244 on CD4^+^ T cells **(C)**. CD244 on CD8^+^ T cells

Supplementary Figure 4
**(A)** Parasitemia and **(B)** Bodyweight curves incl. the absolute number of WT and PDL1 KO mice used per timepoint. Data are from three independent experiments. Values are given as means ± standard error of the means (SEM). **(C)** Tim-3 induction is *T. cruzi* strain independent since it is significantly higher also after infection with the *T. cruzi* Brazil strain, which leads to chronic infections in mice.
