# Supplementary material for: Role of the PD-1/PD-L1 Pathway in Experimental Trypanosoma cruzi Infection and Potential Therapeutic Options
Source: Front Immunol. 2022 Jun 23;13:866120. doi: 10.3389/fimmu.2022.866120 (PMC9260015; doi:10.3389/fimmu.2022.866120)
Supplement: Supplementary Figure 1 — (A) Experimental set-up for the infection of BMDCs in vitro. (B) Flow cytometry gating strategy for BMDCs. [file DataSheet_1.pdf]

S1. Experimental set-up, gating Strategy and exemplary dot blots for PD-L1 expression *in vitro*

A.

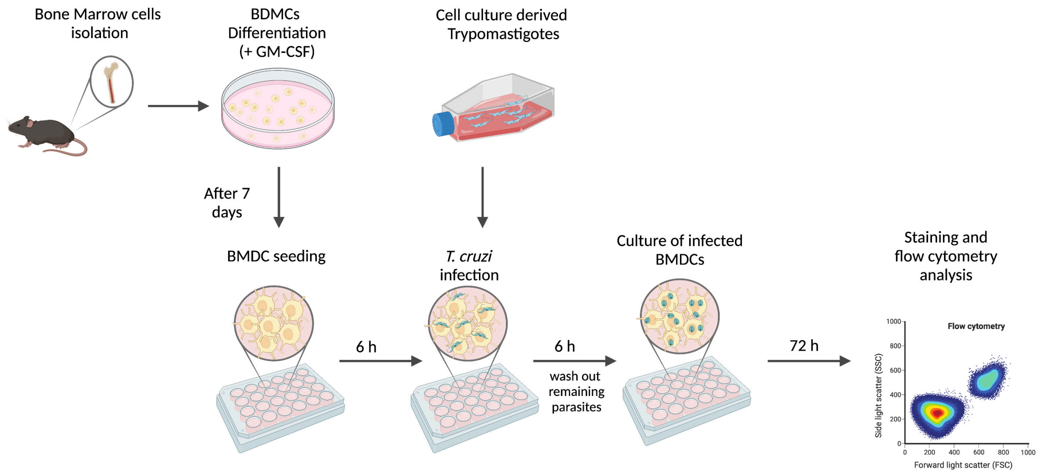

B.

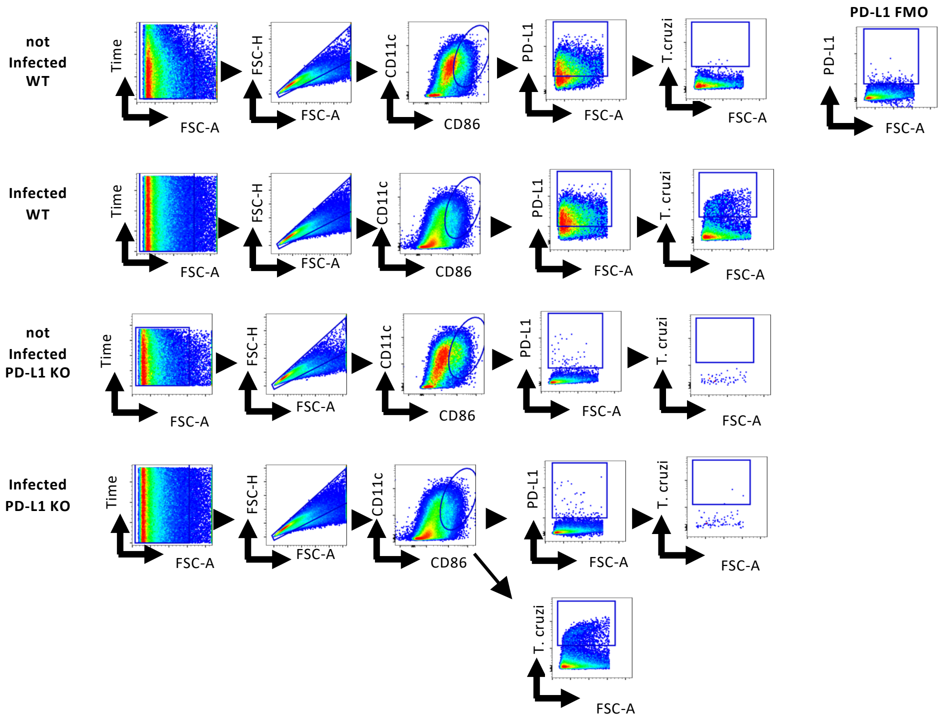

S2. Gating strategy and exemplary dot plots for PD-L1 expression *in vivo*

A.

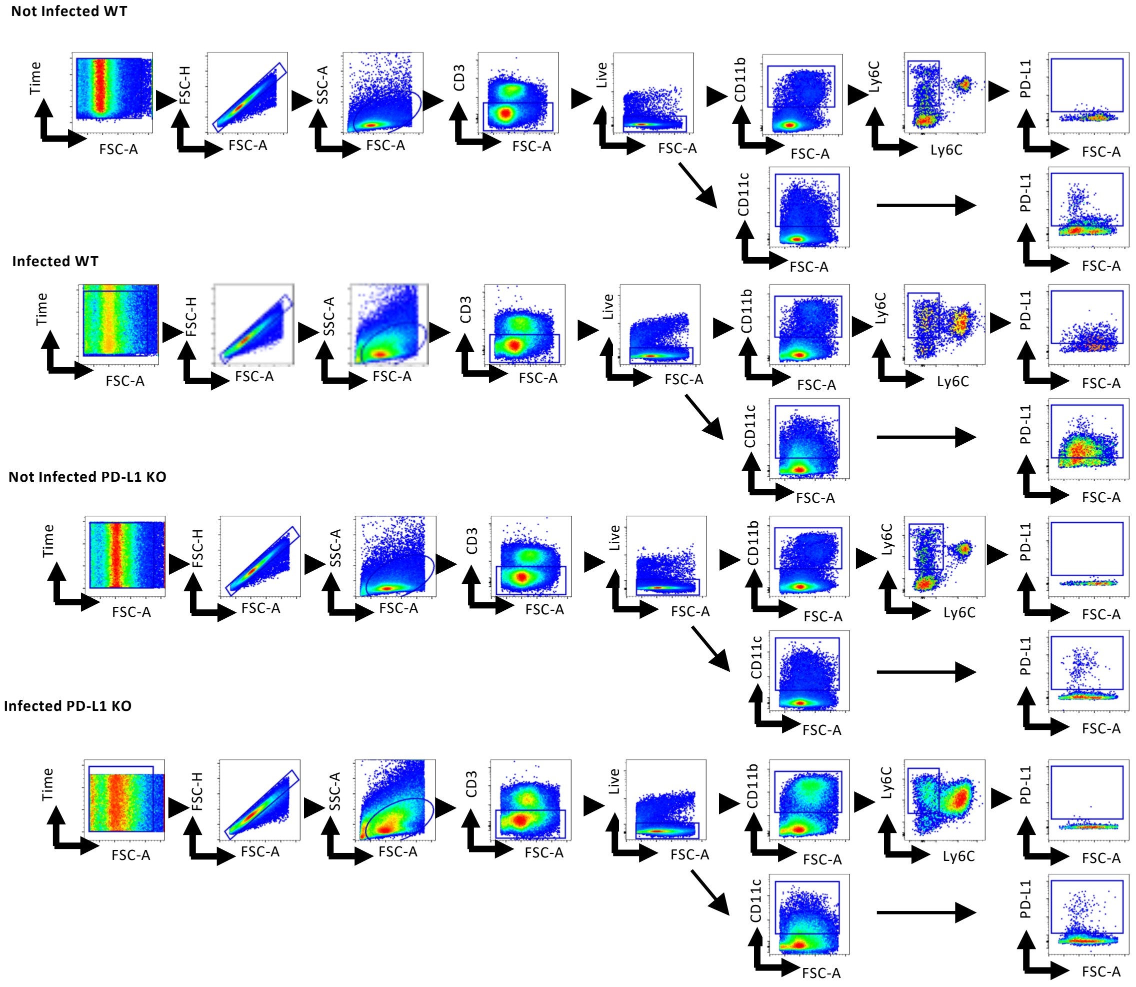

B.

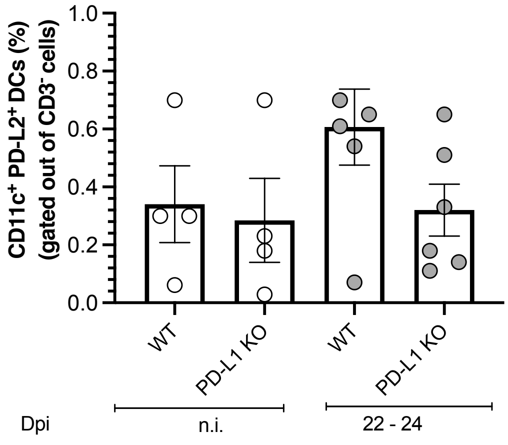

**n**	4	4	6	6
**GM**	0.25	0.17	0.47	0.26

C.

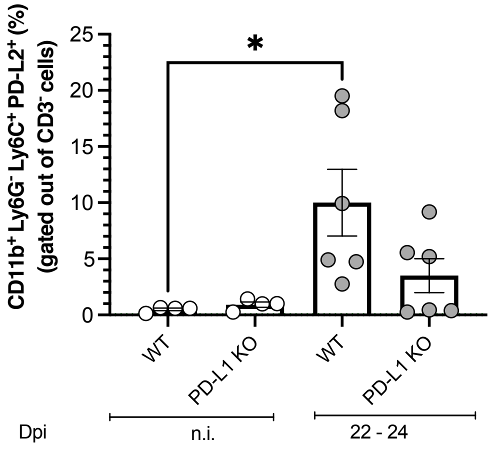

**n**	4	4	6	6
**GM**	0.49	0.91	10	3.5

S3. Gating Strategy and exemplary dot blots for CD4⁺ and CD8⁺ T cells

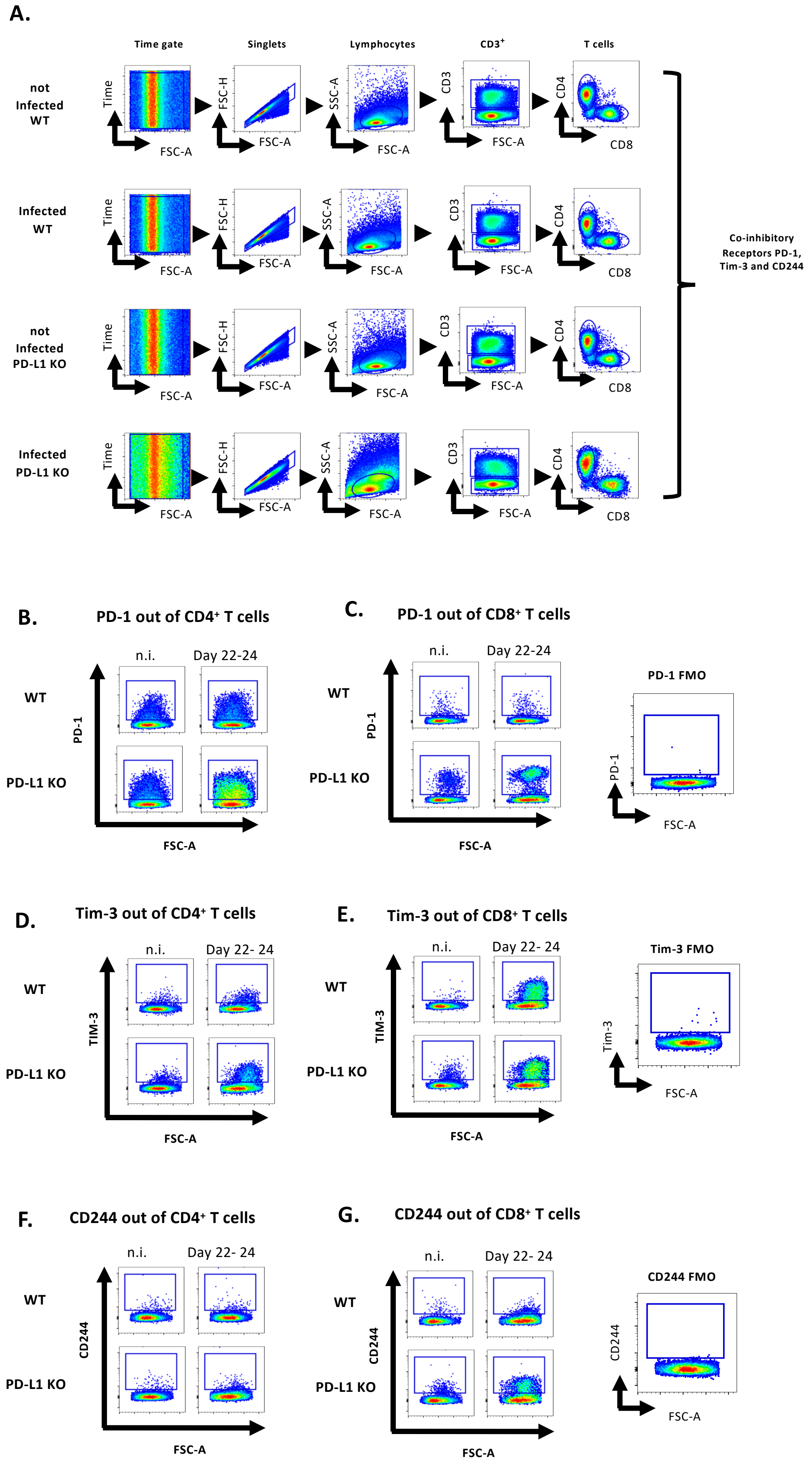

S4.

A.

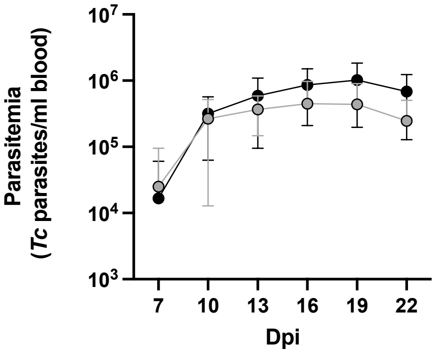

B.

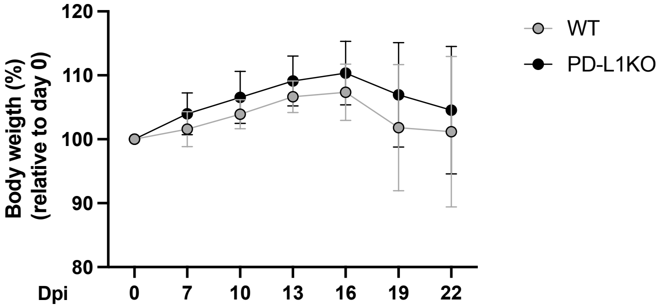

**n**	15	15	15	15	15	14	13
**GM (WT)**	100	101.5	103.8	106.6	107.2	101.38	100.5
**GM (PD-L1KO)**	100	103.9	106.4	109	110.2	106.6	104.1

C.

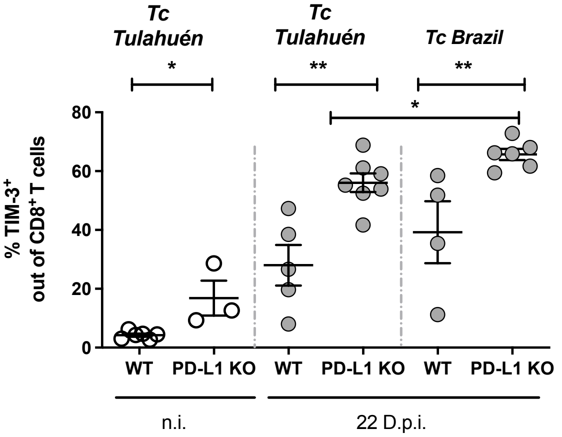

Table 1. List of antibodies used for Flow Cytometry.

Target	Clon	Dilution	Fluorochrome	Manufacturer
CD3	145-2C11	1:200	AF488	Biolegend
CD3	17A2	1:300	Pe/Cy7	Biolegend
CD4	RM4-5	1:400	V500	Biolegend
CD44	IM7	1:400	AF700<br>BV421	Biolegend
PD-L1	10.F9G2	1:200	APC	Biolegend
CD8	53-6.7	1:200<br>1:400	AF700<br>V450	Biolegend<br>eBioscience
2B4 (CD244)	m2B4(B6)458.<br>1	1:200	Pe/Cy7	Biolegeng
PD-1<br>(CD279)	RMP1-30	1:200	PE/Cy7	Biolegend
Tim-3<br>(CD366)	RMT3-23	1:200	APC<br>Pe-Cy7	Biolegend
TNF-α	MP6XT22	1:200<br>1:200	AF700<br>PE	Biolegend<br>Biolegend
IL-10	JESS-16E3	1:100	AF700	eBioscience
Granzyme B	NGZB	1:200	PEeFluor610	eBioscience
CD11c	HL3 RUO	1:200	PE	BD Bioscience
CD86	GL-1			BD Bioscience
PD-L2	TY245	1:100	PE	Biolegend
Donkey anti-<br>Rabbit IgG		1:100	AF488	A-21206
